# Reciprocal-Space Analysis of Structural Reorganization and Orientational Correlation in a Cellulose Nanofibril Suspension during Constant-Stress Creep

**DOI:** 10.1021/acs.langmuir.6c02557

**Published:** 2026-07-24

**Authors:** Yoshifumi Yamagata, Moe Araida, Saki Otobe

**Affiliations:** † Business Unit Characterization, Anton Paar Japan K. K., First fl.,Riverside Sumida, Tsutsumi-Dori 1-19-9, Sumida-ku, Tokyo 131-0034, Japan; ‡ Research Institute for Science and Technology Tokyo University of Science, 2641 Yamazaki, Noda-shi, Chiba 278-8510, Japan

## Abstract

Cellulose nanofibril (CNF) suspensions exhibit yielding and viscoplastic flow because high-aspect-ratio fibrils form entangled networks through fibril entanglement and interfibrillar interactions. However, the time evolution of structural reorganization and orientational correlation under constant stress remains insufficiently understood. In this study, rheo-small-angle light scattering (Rheo-SALS) measurements were performed on a mechanically fibrillated CNF suspension during constant-stress creep at 10, 40, 60, and 200 Pa using parallel-polarized (HH) and cross-polarized (HV) configurations. In the HH mode, at 40–200 Pa, the characteristic correlation length estimated from the shoulder position of the scattering profile decreased sharply during the first 0–20 s and then changed more gradually. The anisotropy ratio over 20–100 s increased with stress and was largest at 200 Pa. In the HV mode, a 4-fold azimuthal pattern developed at 40–200 Pa, with maxima near 45°, 135°, 225°, and 315° and minima near 0°, 90°, 180°, and 270°. The mean peak intensity decreased, the mean trough intensity increased, and an azimuthal modulation index decreased rapidly during 0–20 s, indicating that the major change in orientational correlation was concentrated immediately after stress application. At higher stress, the lower azimuthal modulation index reflected a broader angular distribution of orientational correlation rather than a loss of anisotropy. These results show that the creep response of the CNF suspension under constant stress involves a stress-dependent hierarchical structural transition from an isotropically entangled network to anisotropic mesoscale organization and, at the highest stress, to flow-aligned smaller aggregate units.

## Graphical abstract

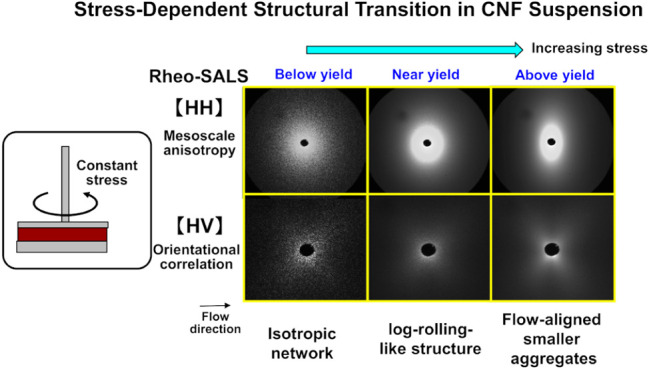

## Introduction

Cellulose nanofibrils (CNFs) have attracted considerable attention in recent years as sustainable biobased nanomaterials because of their high aspect ratio, high strength, low density, and ease of surface functionalization.^1,2^ By taking advantage of these features, CNFs have been increasingly used in a wide range of applications, including coating materials, rheology modifiers, functional composites, and gel-like soft materials.^1,2^ In such applications, the flow properties of CNF suspensions strongly influence both processability and the final material performance. Therefore, a detailed understanding of how the internal structure responds to deformation is important from both fundamental and practical viewpoints.^2–4^

The rheological properties of CNF suspensions are governed by hierarchical and dynamic network structures arising from fibril entanglement, hydrodynamic interactions, and interfibrillar interactions.^3–8^ Accordingly, such systems generally exhibit pronounced shear thinning, viscoelasticity, yielding, and history-dependent responses, indicating that the macroscopic flow properties are strongly coupled with changes in the mesoscale structure under flow.^3,4,6–8^ Complex fluids in which yielding is superimposed on thixotropy and viscoelastic transient responses have been categorized by McKinley, Ewoldt, and coworkers as thixotropic elastoviscoplastic (TEVP) materials.^9,10^ Viewing the constant-stress creep response of a mechanically fibrillated CNF suspension within a TEVP framework is therefore useful for understanding the structural reorganization that occurs immediately after stress application and its subsequent time evolution. However, because the structural information obtained from rheological measurements alone is inherently indirect, it remains difficult to clearly capture when and how fibril orientation, aggregation, and network reorganization occur during deformation, and how these processes contribute to the observed stress response.^4,5^

To address this issue, a method is required that can directly probe the evolving structure under deformation while simultaneously measuring the mechanical response. Small-angle light scattering (SALS) is well suited for probing structural heterogeneity and anisotropy on relatively large length scales in soft materials and dispersed systems, and rheo-small-angle light scattering (Rheo-SALS), which combines SALS with rheology, enables simultaneous measurements of the macroscopic mechanical response and scattering anisotropy.^11–14^ In particular, under constant-stress creep, the rapid deformation immediately after stress application and the subsequent gradual development of deformation are clearly apparent. Rheo-SALS is, therefore, expected to provide a useful means of tracking the corresponding mesoscale structural reorganization and the time evolution of orientational correlation.^13,14^ More broadly, previous rheo-optical and scattering studies on cellulosic materials and related anisotropic soft matter systems have analyzed flow-induced structural evolution using complementary quantities such as the anisotropy of the scattering pattern, azimuthal intensity distributions, and representative structural length scales extracted from radial scattering profiles.^11,13–16,21^ These studies provide a useful framework for interpreting reciprocal-space signatures of collective alignment, structural breakdown and rebuilding, and the emergence of vorticity-related orientational states under flow. Beyond rheo-optical and scattering approaches, combined rheology and complementary in situ characterization techniques have also been developed to correlate macroscopic deformation or flow behavior with the accompanying molecular, mesoscopic, or orientational structural evolution under processing conditions.^22–24^ For example, integrated rheology/spectroscopy methods, including rheology coupled with infrared or Raman spectroscopy, have been used to monitor flow-induced aggregation, shear-induced alignment, and flow-induced crystallization in soft and polymeric systems.^22–24^ These studies demonstrate the broader value of hybrid methods that couple rheology to spectroscopic or structural probes. The present Rheo-SALS approach should, therefore, be viewed within this broader class of combined rheology/structure characterization techniques while being specifically suited to reciprocal-space analysis of characteristic length scale, mesoscale anisotropy, and orientational correlation in CNF suspensions during deformation.

Previous studies have investigated the relationships between the structure and flow properties of CNF suspensions using steady shear measurements, dynamic viscoelastic measurements, microscopic observations, polarized optical observations, and various scattering methods.^3–6,13,15^ These studies have shown that, depending on the concentration, aspect ratio, surface chemistry, and ionic environment, the dispersion state of the fibrils and the network structure change, and the flow curves and viscoelastic responses vary accordingly.^4–6^ More recently, the present authors reported, using the Rheo-Iris polarized imaging technique, the correspondence among the yielding transition, viscoplastic flow, and two-dimensional microstructural changes during the constant-stress creep process of a mechanically fibrillated CNF suspension.^16,17^ However, because that previous study relied mainly on birefringence images and the spatial distribution of orientation axes in real space, it was not sufficient to quantitatively discuss changes in the representative length scale and the time evolution of orientational correlation in reciprocal space.

Against this background, in the present study, rheo-SALS measurements were performed on a mechanically fibrillated CNF suspension to analyze the time evolution of structural reorganization and orientational correlation under constant-stress creep. Specifically, scattering patterns were recorded in parallel-polarized (HH) and cross-polarized (HV) configurations. The HH scattering was used to evaluate the characteristic correlation length and its anisotropy, whereas the HV scattering was used to evaluate the 4-fold orientational correlation and changes in angular modulation. Through this approach, we aimed to clarify the time window over which the CNF network undergoes rapid reorganization and the nature of the anisotropic mesoscale structures that subsequently develop under stress conditions ranging from below yielding to above yielding. In addition, this study extends the real-space observations reported in the previous Rheo-Iris study from the viewpoint of scattering anisotropy, thereby providing a more comprehensive understanding of the process-structure–property relationships in mechanically fibrillated CNF suspensions.^16^ More specifically, by linking the imposed stress level and creep time history to reciprocal-space measures of characteristic length and orientational correlation, the present approach makes it possible to identify which processing conditions preserve an isotropic entangled network, promote the formation of anisotropic mesoscale structures near yielding, or induce flow-aligned smaller aggregate units at higher stress. Such information is directly relevant to process-structure–property relationships because these stress-dependent structural pathways govern the macroscopic creep response and the development of flow-induced anisotropy in CNF suspensions and their derived materials. From a processing viewpoint, this information is relevant to operations such as pumping, mixing, coating, and flow-assisted forming, in which the imposed stress and its time history determine how the CNF network reorganizes under deformation. From an application viewpoint, these structurally distinct states are expected to influence not only the transient flowability of the suspension but also the anisotropy, uniformity, and potentially the performance of CNF-based materials formed during or after processing.

In this paper, we first identify the apparent yielding regime of the CNF suspension and define the stress regimes examined in this study on the basis of strain-amplitude sweep measurements and constant-stress creep measurements. We then discuss the reorganization and anisotropization of the mesoscale structure under constant stress based on the HH scattering analysis and clarify the formation of orientational correlation and its time evolution based on the HV scattering analysis. Finally, these results are interpreted together with the polarized imaging results obtained in the previous Rheo-Iris study, and the hierarchical structural transition during the creep process of the mechanically fibrillated CNF suspension is discussed.

## Experimental Methods

### Sample Preparation

The CNF suspension used in this study was the same extra-long type (IMa-10002, Sugino Machine Co., Ltd.) as that used in our previous study.^16^ The suspension was supplied as a 2 wt % aqueous dispersion and was produced by mechanical fibrillation using a water-jet process. No dilution was performed, and the suspension was used as received without any additional purification. Before measurement, the sample was stored under refrigerated conditions. Prior to the rheological and Rheo-SALS measurements, the suspension was removed from refrigeration and allowed to equilibrate at room temperature (ca. 20–25 °C). It was then loaded onto the rheometer, and all measurements were performed at 20 °C. In the present proof-of-concept study, the CNF concentration and measurement temperature were not varied; instead, a single representative condition, namely the as-received 2 wt % suspension measured at 20 °C, was used to focus on the stress-dependent time evolution of the Rheo-SALS response under well-defined experimental conditions.

### Strain–Amplitude Sweep Measurements

To clarify the relation of the selected constant-stress creep conditions to the apparent yielding regime of the CNF suspension, strain-amplitude sweep measurements were performed. A stress-controlled rheometer (MCR302e, Anton Paar GmbH, Graz, Austria), equipped with a 40 mm parallel-plate geometry, was used. The gap was set to 0.5 mm, the angular frequency was fixed at 10 rad s^–1^, and the strain amplitude was swept. From the resulting storage modulus *G*′, loss modulus *G*″, and the stress–strain amplitude relationship, the linear viscoelastic region and the apparent yielding regime were identified. On this basis, the constant-stress creep and Rheo-SALS measurements described below were performed under representative stress conditions: below yielding, near yielding, and above yielding.

### Rheo–Small-Angle Light Scattering (Rheo-SALS) Measurements

Figure 1 shows a schematic illustration of the Rheo-SALS setup. A laser source (λ = 658 nm) was mounted above the rheometer described above, and the laser beam was introduced parallel to the rotational axis. The transmitted scattering light was projected onto a screen and recorded by a CCD camera. The camera length, *l*, was 150 mm, and the beam stopper diameter was 1 mm. The measurement fixture consisted of an upper quartz plate with a diameter of 43 mm and a lower quartz plate separated by a gap of 0.5 mm. After the upper plate had reached the measurement position, the sample was left undisturbed for 2 min to minimize any preload before the start of the measurement. Although the preload was not quantitatively confirmed to be exactly zero, no intentional preload was applied during the subsequent measurement. Constant stresses of 10, 40, 60, and 200 Pa were applied to the sample for 120 s, and the time evolution of strain and the scattering patterns for the selected polarization configuration were recorded simultaneously. The stress was applied as a programmed step in the rheometer-controlled constant-stress mode, although the exact subsecond rise time was not independently quantified in the present study. The creep strain data shown in Figure 3 were recorded by the rheometer from 0.1 s after stress application, and six data points were obtained within the first 1 s. The scattering measurements in the parallel-polarized configuration (HH) and a cross-polarized configuration (HV) were performed separately in independent experiments, where H and V denote horizontal and vertical, respectively. During each experiment, scattering images were acquired every 20 s. The exposure time for each image was 10.6 ms for HH and 55 ms for HV. The camera gain was fixed at 1.0 in both modes, and no additional corrections, such as transmission normalization, absolute-intensity calibration, detector-sensitivity correction, or explicit background subtraction, were applied to the recorded scattering intensities. Therefore, the scattering intensities were analyzed comparatively within each polarization mode, and no direct absolute comparison was made between the HH and HV modes. All measurements, including the strain-amplitude sweep measurements, were carried out at 20 °C under conditions that suppressed solvent evaporation. For the quantitative analysis, only the data acquired during constant stress application (0–100 s) were used, whereas the images recorded at 120 s were excluded because they included structural relaxation immediately after cessation of the applied stress. Compared with the corresponding scattering patterns at 100 s, the 120 s patterns showed a decrease in overall scattering intensity and a change in pattern shape toward the prestress state, indicating the onset of structural relaxation.

**Figure 1:**
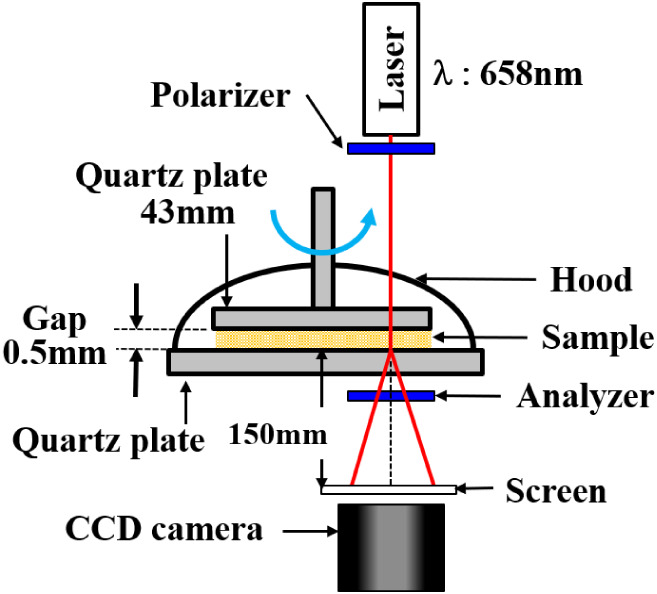
Schematic illustration of the experimental setup for rheo–small-angle light scattering (Rheo-SALS). The sample was confined between an upper rotating quartz plate (43 mm diameter) and a lower fixed quartz plate with a gap of 0.5 mm. Shear stress was applied through the rotation of the upper plate.

### Data Analysis of SALS Patterns

Scattering profiles in the 0° direction, corresponding to the flow direction, and in the perpendicular 90° direction were extracted from the HH scattering patterns. Because the HH scattering profiles contained a small water-derived background, which was observed mainly in the low-q region, a characteristic scattering vector, *q*∗, was determined from the shoulder position of the scattering profile. The direct beam was blocked by a 1 mm-diameter beam stopper, and the central region of each scattering pattern was therefore masked and excluded from the extraction of the 1D profiles. Accordingly, only q values above the effective lower limit of the accessible range were used in the analysis, and the very low-*q* turnover was not used for quantitative interpretation. No explicit background subtraction was applied, because the scattering from the CNF suspension was substantially stronger than the water contribution, and the latter was judged to be small enough not to affect the present analysis seriously. Specifically, the low-*q* and high-*q* regions of each scattering profile on the log–log plot were approximated by straight lines, and their intersection was defined as *q*∗. Although this procedure is conceptually related to an empirical two-power-law description of the scattering profile, the linear fits were used here only as local operational approximations to determine the shoulder position in a simple and consistent manner. No full power-law analysis or fitting of the entire profile to a specific empirical function was attempted, because the aim of the present study was to track the time evolution of q* and the corresponding characteristic correlation length, ξ, under constant-stress creep. For each stress and azimuthal direction, the *q* ranges used for fitting were kept identical for all time points, and data points potentially affected by detector saturation in the lowest-*q* region were excluded from the fitting. The fitting ranges are listed in Table S1, and the resulting *q*∗ values are summarized in Table S2. The characteristic correlation length, ξ, was operationally estimated from *q*∗ on the basis of a Bragg-type reciprocal-space relation^18^ as follows:

1 ξ=2πq*

Here, ξ is treated not as the actual size of an individual CNF aggregate itself, but as a representative length reflecting the inhomogeneity or density correlation of the CNF network. To quantify the directional structural anisotropy, the average characteristic correlation lengths in the 0° and 90° directions over 20–100 s were denoted as ξ_0°_ and ξ_90°_, respectively, and the anisotropy ratio, A_ξ_, was defined as

2 Aξ=ξ0°ξ90°

This ratio was adopted as a simple dimensionless measure of the directional anisotropy of the representative structural length scale extracted from the HH scattering profiles. With this definition, A_ξ_ = 1 corresponds to an isotropic state, whereas deviations from unity indicate the development of directional structural anisotropy between the flow and perpendicular directions. Other scalar definitions, such as a simple difference or a normalized difference between ξ_0°_ and ξ_90°_, are also possible, but the ratio form was used here because it enables direct comparison among stress conditions independent of the absolute magnitude of ξ. In the present HH scattering analysis, this ratio was introduced to evaluate the directional anisotropy of the representative structural length scale along the flow (0°) and perpendicular (90°) directions, rather than to define a pure orientational order parameter from the full azimuthal intensity distribution on the detector. Accordingly, Aξ should be interpreted as an operational measure of directional structural anisotropy and may reflect not only orientation of the correlated structures but also the directional deformation of the scattering feature under stress.

The present analysis combines radial and azimuthal evaluations, in line with common reciprocal-space approaches used in rheo-optical and SALS studies of anisotropic soft materials and cellulosic suspensions, in which representative structural length scales are evaluated from the radial features of the scattering profile, and directional anisotropy is quantified from the azimuthal intensity distribution.^11,13,14,21^ In this sense, the HH and HV data were treated complementarily in the present study: the shoulder position in the HH scattering profiles was used to evaluate the direction-dependent characteristic correlation length, whereas the azimuthal intensity distribution of the HV 4-fold pattern was used to discuss orientational correlation more directly. In the present work, the shoulder position in the HH scattering profiles and the peak-to-trough contrast of the HV 4-fold pattern were adopted as operational quantities suited to the observed data set.

The azimuthal intensity distribution, *I*(χ), was extracted from an annulus at *q* = 1.88 ± 0.04 μm^–1^ (900 ± 5 pixels), where the four scattering peaks were most clearly separated, and the effects of low-*q* saturation and the vicinity of the beam stopper could be avoided. For consistency of comparison, the same *q* range was used for all stresses and all time points. For the HV scattering, no explicit background subtraction was performed because the solvent contribution was judged to be sufficiently small in the cross-polarized configuration. Indeed, scattering from water alone was hardly detectable in the HV configuration, and the recorded HV intensities were therefore regarded as being dominated by the sample contribution. The average intensity at 45°, 135°, 225°, and 315° was defined as the mean peak intensity, *I̅_max_*, whereas the average intensity at 0°, 90°, 180°, and 270° was defined as the mean trough intensity, *I̅_min_*. These quantities were both evaluated at fixed angular positions on the selected annulus at *q* = 1.88 ± 0.04 μm^–1^ and therefore represent local mean intensities at those nominal peak and trough directions, rather than integrated intensities of the full azimuthal lobes over their entire angular widths. Although several order parameters could, in principle, be used to quantify 4-fold anisotropy, in the present study, we adopted C­(*t*) as a simple azimuthal modulation index. Because the HV scattering patterns exhibited stable maxima near 45°, 135°, 225°, and 315° and minima near 0°, 90°, 180°, and 270° throughout the measurement, the normalized peak-to-trough difference provides a direct measure of the amplitude of the 4-fold angular modulation at the selected *q*. This definition was chosen as an operational metric that does not require fitting the full azimuthal profile to a specific functional form and, by averaging over the four maxima and four minima, reduces the influence of local noise and slight asymmetry among the peaks. In the present data, the four peak intensities were not always perfectly identical, and two opposite peaks could be slightly higher than the other pair. Such small deviations from ideal 4-fold symmetry were not analyzed separately in the present study, because the objective was to quantify the overall 4-fold modulation. Here, C­(*t*) is not intended to represent scattering contrast arising from refractive-index differences between scatterers, nor is it equivalent to a scattering invariant. Rather, it is a local, dimensionless measure of the angular modulation of the HV intensity distribution at the selected *q*. On this basis, the azimuthal modulation index, C­(*t*), was defined as

3 $$C\left(t\right)=\frac{\left({\overset{-}{I}}_{\max}‐{\overset{-}{I}}_{\min}\right)}{\left({\overset{-}{I}}_{\max}+{\overset{-}{I}}_{\min}\right)}$$

The quantitative analyses of both the HH and HV scattering were performed using only the data obtained during constant stress application (0–100 s). The scattering patterns recorded at 120 s were excluded from the quantitative analysis because they included structural relaxation immediately after cessation of the applied stress.

## Results and Discussion

### Strain-Amplitude Sweep and Creep Measurements of the CNF Suspension

To clarify the apparent yielding regime and place the selected constant-stress creep conditions relative to it, strain-amplitude sweep measurements were first performed on the CNF suspension. As shown in Figure 2a, both the storage modulus, *G*′ and the loss modulus, *G*″ remained nearly constant in the low-strain region up to a strain amplitude, γ, of 2.5 × 10^–2^ (2.5%), indicating the linear viscoelastic region (LVER), and in this region, *G*′ was larger than *G*″. Both moduli then decreased with increasing γ, and eventually, the magnitude relationship between *G*′ and *G*″ was reversed. When the stress, σ, was plotted against the strain amplitude, γ, from the strain-sweep measurement, as shown in Figure 2b, the stress initially increased linearly with strain up to approximately 10 Pa, after which it deviated from the line and a plateau-like region appeared around 40–60 Pa. This plateau-like region was regarded as the apparent yielding regime.^19,20^ On this basis, the four stress conditions used in this study were defined as representative conditions below yielding (10 Pa), near yielding (40 and 60 Pa), and above yielding (200 Pa).

**Figure 2:**
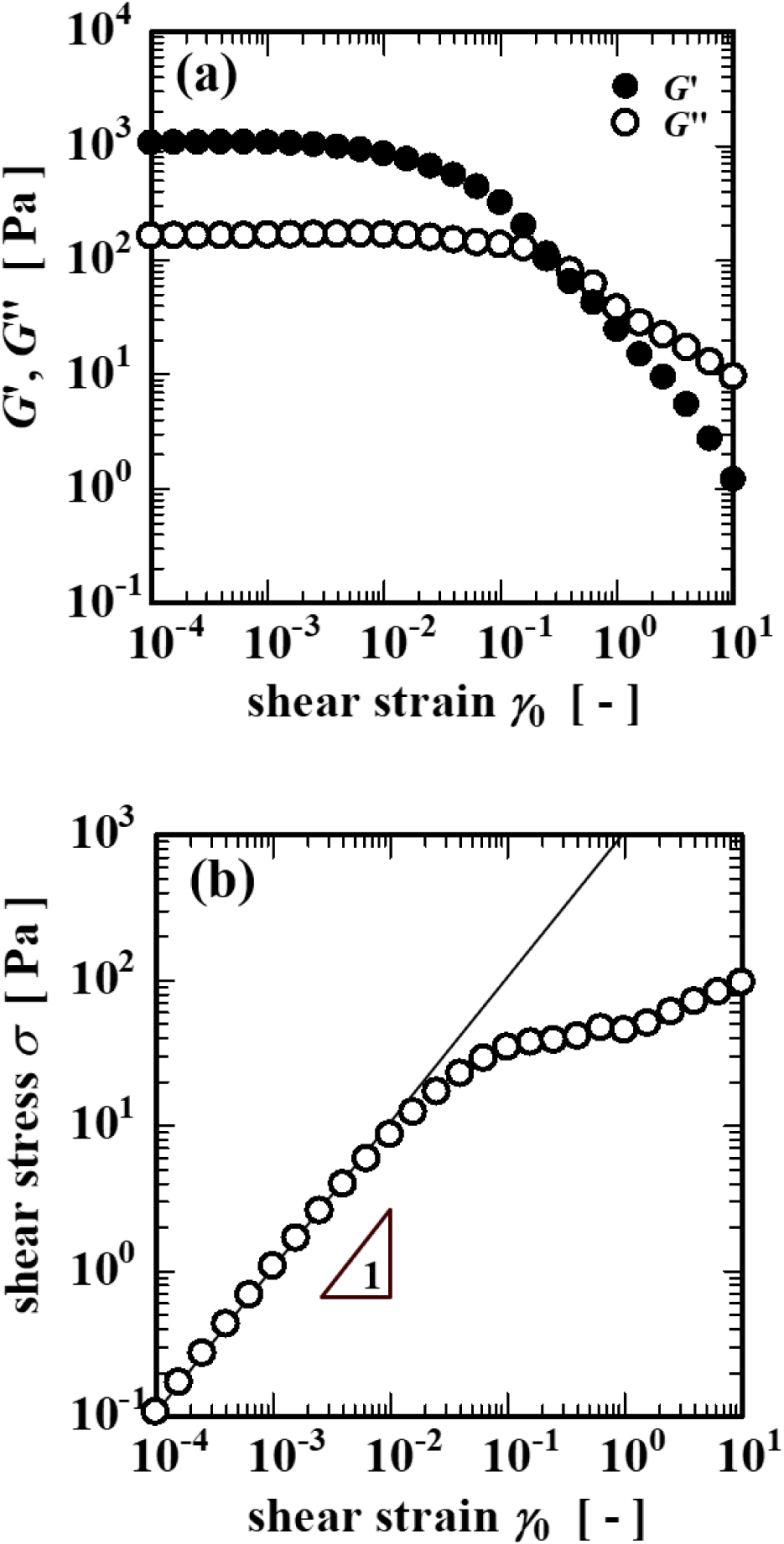
Rheological characterization of the CNF suspension obtained from strain-amplitude sweep measurements: (a) storage modulus *G*′ and loss modulus *G*″ as functions of dimensionless strain amplitude at an angular frequency of 10 rad s^–1^; (b) shear stress–strain amplitude curve derived from the strain-amplitude sweep data. The plateau-like region around 40–60 Pa in panel (b) was regarded as the apparent yielding regime.

Based on this classification, creep measurements were conducted under four stress conditions spanning the preyield, near-yield, and postyield regimes. The resulting creep curves are shown in Figure 3a, and the corresponding log–log representation is shown in Figure 3b. Under all applied stresses, the strain γ increased rapidly immediately after stress application. As shown more clearly in Figure 3b, the response then transitioned to a later-time regime. For 40–200 Pa, the later-time behavior approached an approximately linear increase in strain with time, although the slope remained slightly larger than 1 within the present observation window. At 10 Pa, the increase in strain remained much smaller throughout the measurement.

**Figure 3:**
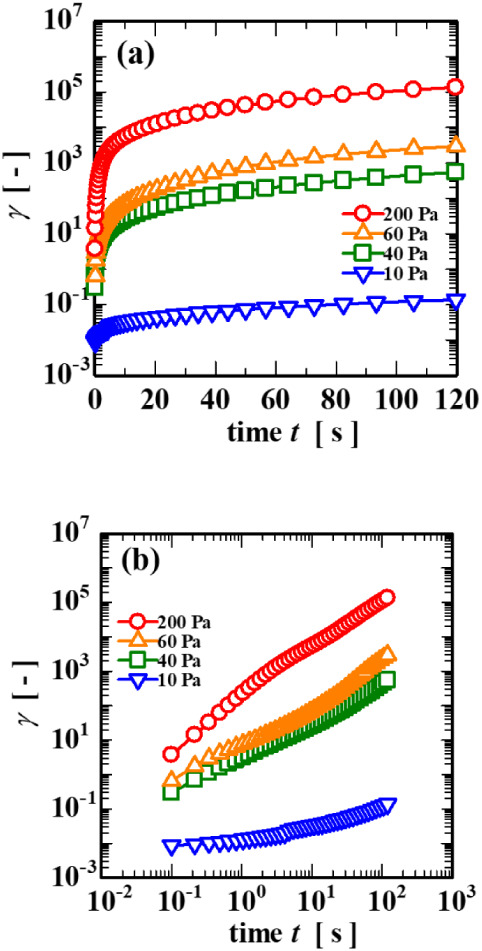
Creep curves of the CNF suspension under constant shear stresses of 10, 40, 60, and 200 Pa are shown as (a) dimensionless strain versus time on a linear scale and (b) the corresponding log–log plot. The log–log representation more clearly shows the transition from the initial rapid increase in strain to a later-time regime. For 40–200 Pa, the later-time behavior approaches an approximately linear increase in strain with time, although the slope remains slightly larger than 1 within the present observation window.

### Structural Changes Under Constant Stress Revealed by HH Scattering

Figure 4 shows a series of HH scattering patterns of the CNF suspension extracted at 20 s intervals under constant stress. Although the constant stress was applied up to 120 s, the scattering pattern recorded at 120 s included the effect of structural relaxation associated with stress cessation. Compared with the corresponding pattern at 100 s, it showed a lower overall scattering intensity and a pattern shape closer to that before stress application, indicating the onset of structural relaxation. Therefore, the quantitative analysis of the HH scattering was limited to the data obtained over 0–100 s, which reflect the structure under constant stress. At 10 Pa, no pronounced change was visually discernible in the 2D scattering patterns throughout the measurement, and an almost isotropic scattering pattern was maintained. In contrast, at 40, 60, and 200 Pa, the scattering patterns clearly changed within 20 s after stress application, and anisotropic scattering patterns were maintained thereafter up to 100 s. In particular, at 200 Pa, the anisotropy of the scattering pattern became most pronounced after 20 s, suggesting that under high stress the CNF network evolves into a structure with a preferred direction in response to the flow field. Thus, the 2D HH images indicate that the major visible structural response occurred within the first 20 s after stress application.

**Figure 4:**
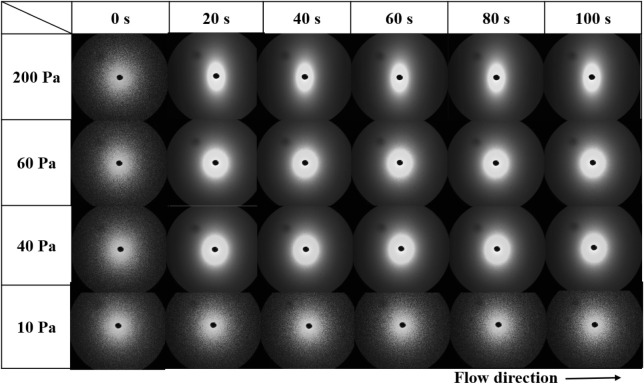
Time evolution of the HH scattering patterns of the CNF suspension under constant shear stresses of 10, 40, 60, and 200 Pa. The images shown were recorded at 0, 20, 40, 60, 80, and 100 s after stress application. The flow direction is from left to right. Although the stress was applied up to 120 s, the image at 120 s was excluded from the quantitative analysis because it was recorded immediately after stress cessation and showed a decrease in scattering intensity together with a change in pattern shape toward the prestress state.

Figure 5 shows the scattering profiles in the 0° and 90° directions extracted from the HH scattering patterns in Figure 4. These scattering profiles include the water background. At 10 Pa, the changes in the profile shape were relatively small in both the 0° and 90° directions, and the time-dependent change in the shoulder position was also limited. In contrast, at 40, 60, and 200 Pa, the shoulder position shifted toward the higher-*q* side from 0 to 20 s, after which it remained nearly constant or changed only slightly. In other words, the representative length scale reflected in the scattering decreased rapidly within the first 20 s after stress application and then approached a nearly constant value. In addition, at 200 Pa, the difference in the profile shape, namely the shoulder position, between the 0° and 90° directions became more evident, indicating that the structural anisotropy becomes more pronounced at higher stress.

**Figure 5:**
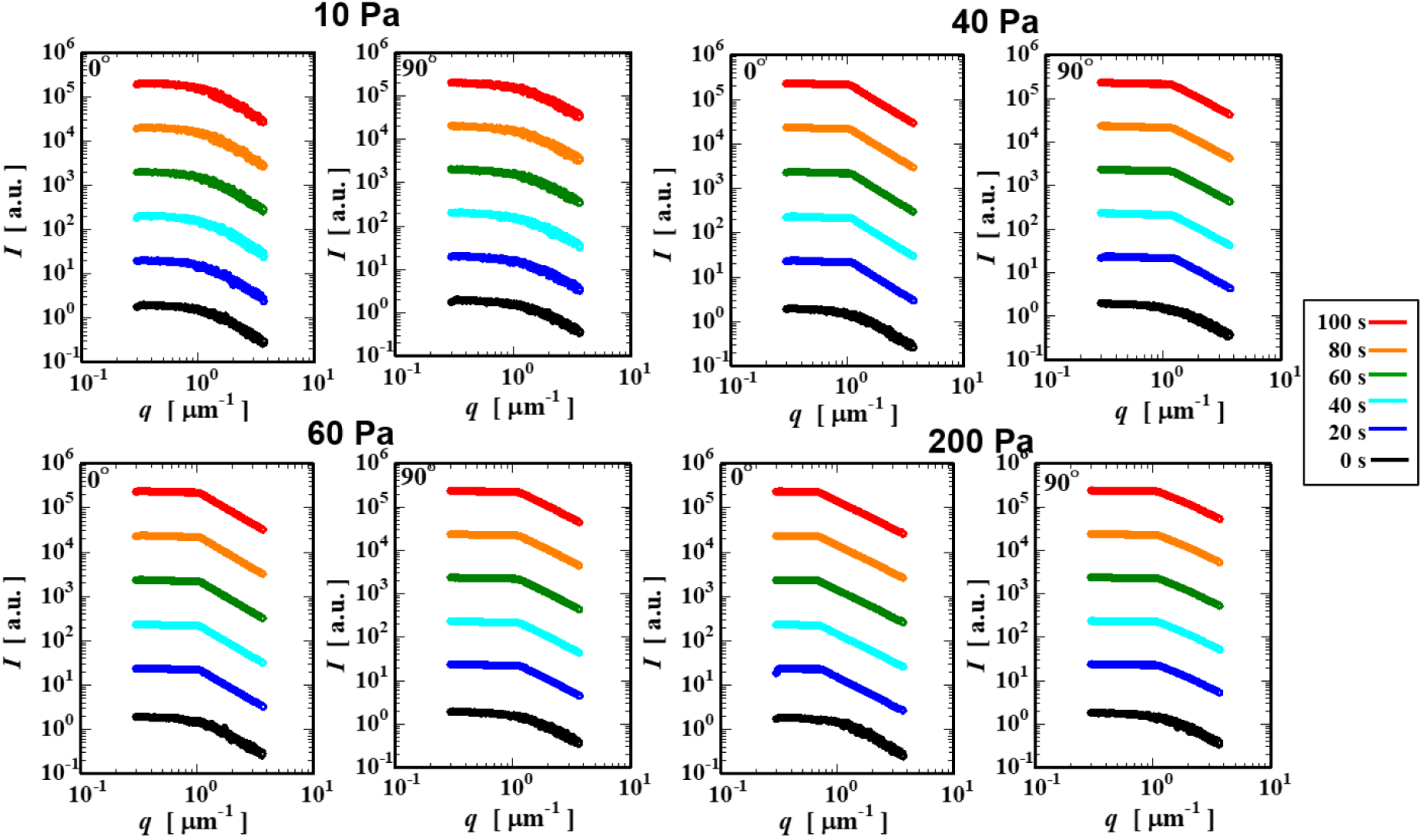
Time evolution of the HH scattering profiles of the CNF suspension in the 0° and 90° directions under constant shear stresses of 10, 40, 60, and 200 Pa, extracted from the HH scattering patterns shown in Figure 4. The scattering profiles include the water background. The characteristic scattering vector, *q*∗, was defined from the shoulder position of the scattering profile. Only the data obtained under constant stress (0–100 s) were used for the quantitative analysis. The direct beam was blocked by a 1 mm-diameter beam stopper, and the lowest-*q* region was therefore masked and excluded from the analysis; accordingly, the effective lower limit of the accessible q range was approximately 0.17 μm^–1^, corresponding to a maximum real-space length scale of about 60 μm.

Next, the scattering vector at the shoulder position of the scattering profile was defined as *q*∗, and the characteristic correlation length, ξ, was calculated from eq 1 and Figure 6 shows the time evolution of ξ. At 40, 60, and 200 Pa, ξ decreased sharply from 0 to 20 s in both the 0° direction (Figure 6a) and the 90° direction (Figure 6b), and then remained nearly constant or changed only slightly over 20–100 s. For example, in the 0° direction, ξ decreased from approximately 10.9 to 5.9 at 40 Pa, from approximately 9.1 to 6.3 at 60 Pa, and from approximately 11.5 to 8.6 at 200 Pa. In contrast, at 10 Pa, the magnitude of the change was relatively small, and no major structural change was observed throughout the measurement. The ξ obtained here does not represent the actual size of the CNF aggregates themselves, but rather a characteristic length reflecting the inhomogeneity or density correlation of the CNF network. Therefore, the sharp decrease in ξ during 0–20 s suggests that the coarse isotropic network formed at rest rapidly collapsed and rearranged immediately after stress application, leading to a flow-induced structure on a smaller length scale.^14^ This interpretation is also consistent with the creep curves, which showed a rapid increase in strain immediately after stress application, followed by a later-time regime in which the rate of change became much smaller.

**Figure 6:**
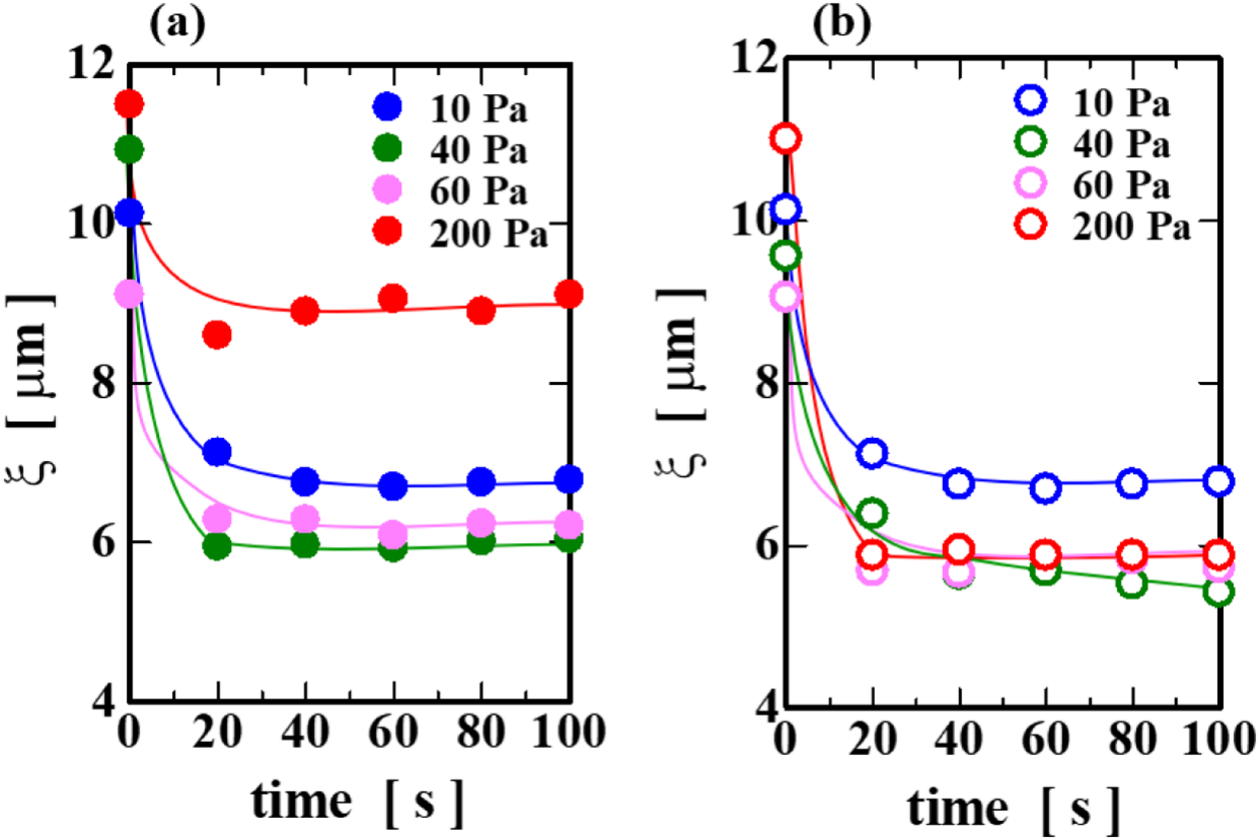
Time evolution of the characteristic correlation length, ξ, calculated as 2*π*/*q* ^*^ from the HH scattering profiles shown in Figure 5 for the (a) 0° and (b) 90° directions. The analysis was performed using the data obtained during constant stress application (0–100 s). The lines are guides to the eye and do not represent fits to any specific model or function.

After 20 s, the directional anisotropy quantified by the anisotropy ratio A_ξ_ = ξ_0°_/ξ_90°_ became more informative than the absolute value of ξ itself. Figure 7 summarizes the stress dependence of A_ξ_ using the average characteristic correlation lengths over 20–100 s, i.e., after the initial transient decrease in ξ. This comparison was made on a common time basis under constant-stress creep to evaluate the anisotropy sustained under each applied stress during the post-transient regime. At 10 Pa, the ξ values in the two directions were nearly identical, indicating that the structure remained almost isotropic. In contrast, A_ξ_ slightly exceeded 1 at 40 and 60 Pa and increased to approximately 1.5 at 200 Pa. Thus, whereas structural anisotropy was scarcely generated at 10 Pa below yielding, anisotropy emerged at stresses near and above yielding and became most pronounced at 200 Pa.

**Figure 7:**
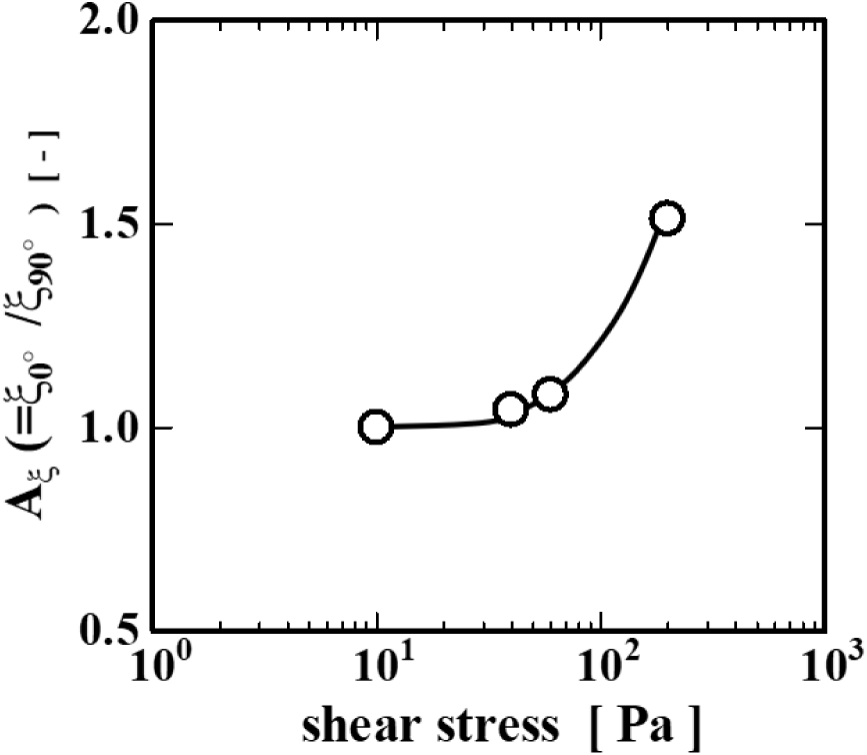
Stress dependence of the anisotropy ratio of the characteristic correlation length, $A_{\xi }=\xi_{0{}^{\circ}}/\xi_{90{}^{\circ}}$ , where ξ_0°_ and ξ_90°_ are the average characteristic correlation lengths over 20–100 s in the 0° and 90° directions, respectively. A_ξ_ was used here as a simple dimensionless measure of the directional anisotropy of the representative structural length scale extracted from the HH scattering profiles; A_ξ_ = 1 corresponds to an isotropic state, whereas deviations from unity indicate the development of directional structural anisotropy between the flow and perpendicular directions. The increase in A_ξ_ with applied stress indicates enhanced anisotropic restructuring of the CNF network under higher constant stresses.

Overall, the HH scattering analysis revealed that, under constant-stress creep, the characteristic length of the mesoscale structure in the CNF suspension decreased sharply immediately after stress application, followed by the development of a more pronounced directional anisotropy at higher stress. Thus, time-resolved HH scattering by Rheo-SALS provides a useful reciprocal-space measure of the stress-dependent structural reorganization of the CNF network under the present constant-stress creep conditions.

### Structural Changes Under Constant Stress Revealed by HV Scattering

Figure 8 shows the time evolution of the HV scattering patterns of the CNF suspension under a constant stress. The HV scattering was acquired in the cross-polarized configuration (HV), which makes it possible to emphasize the anisotropic scattering component arising from the orientational correlation of the CNF network. At 10 Pa, no pronounced change was visually discernible in the scattering pattern throughout the measurement, and the development of a distinct anisotropic scattering pattern was limited. In contrast, at 40, only weak but discernible changes appeared after stress application, whereas at 60 and 200 Pa, the changes were much more evident. The region where these poststress changes are most clearly observed is highlighted in Figure 8. At 40, 60, and 200 Pa, a 4-fold-symmetric scattering pattern became discernible after 20 s, although at 40 Pa this anisotropy was much weaker than at 60 and 200 Pa. In particular, at 200 Pa, the 4-fold-symmetric pattern became most distinct after 20 s, suggesting that an anisotropic structure accompanied by orientational correlation was formed in the CNF network under high stress. These results indicate that the HV scattering visualizes the time evolution of orientational correlation developing under flow from a viewpoint different from that of HH scattering.

**Figure 8:**
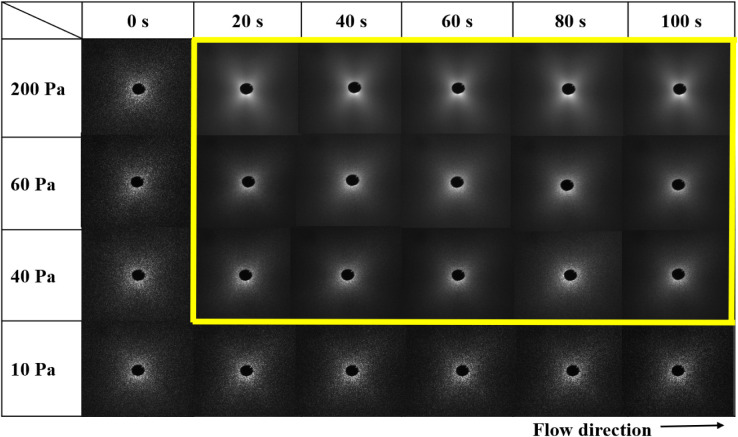
Time evolution of the HV scattering patterns of the CNF suspension under constant shear stresses of 10, 40, 60, and 200 Pa. The images shown were recorded at 0, 20, 40, 60, 80, and 100 s after stress application. The flow direction is from left to right. The highlighted region indicates where the poststress changes in the scattering pattern are most clearly observed for 40–200 Pa.

Figure 9 shows the azimuthal intensity distributions, *I*(χ), obtained from the HV scattering patterns in Figure 8. Comparison of the angular distributions at 0, 20, and 100 s shows that, at 40, 60, and 200 Pa, intensity maxima appeared near 45°, 135°, 225°, and 315°, whereas intensity minima appeared near 0°, 90°, 180°, and 270°, demonstrating that the HV scattering exhibited a 4-fold-symmetric anisotropy. Although the four maxima were not always perfectly identical in height, the dominant feature was still the emergence of the 4-fold angular modulation. The small difference between the two peak pairs likely reflects a slight residual asymmetry in the optical system and/or the sheared sample, rather than a change in the principal angular positions of the pattern. At 40 Pa, however, this modulation was still weak and should be regarded as only partially developed, whereas it became much more evident at 60 and 200 Pa. At 10 Pa, similar intensity modulations at the corresponding angular positions were also observed, but the time dependence was smaller than that at 40–200 Pa. Therefore, at stresses near and above yielding, a 4-fold-symmetric scattering pattern reflecting orientational correlation was rapidly formed immediately after stress application. In addition, at 40–200 Pa, the angular positions of the maxima and minima did not change appreciably throughout the measurement, suggesting that the angular intensity modulation changed while the principal axes were maintained, rather than that the principal axes themselves rotated. Because the quantitative analysis uses averages over the four nominal peak directions and the four nominal trough directions, these small peak-to-peak differences do not affect the main interpretation of the HV scattering evolution. The *I*(χ) profile at 10 Pa should be treated as a reference condition that is susceptible to the influence of a low signal-to-noise ratio because both the absolute intensity and its time variation were small. The noisier appearance of the azimuthal profiles at lower stresses is attributed to a lower signal-to-noise ratio in the HV scattering, arising from the weaker scattered intensity under these conditions. To make the definitions used in the quantitative analysis explicit, the representative 100 s profile in Figure 9d is annotated with downward green arrows indicating the peak directions at 45°, 135°, 225°, and 315°, and upward pink arrows indicating the trough directions at 0°, 90°, 180°, and 270°. These angular positions were fixed throughout the analysis for all stress conditions and time points.

**Figure 9:**
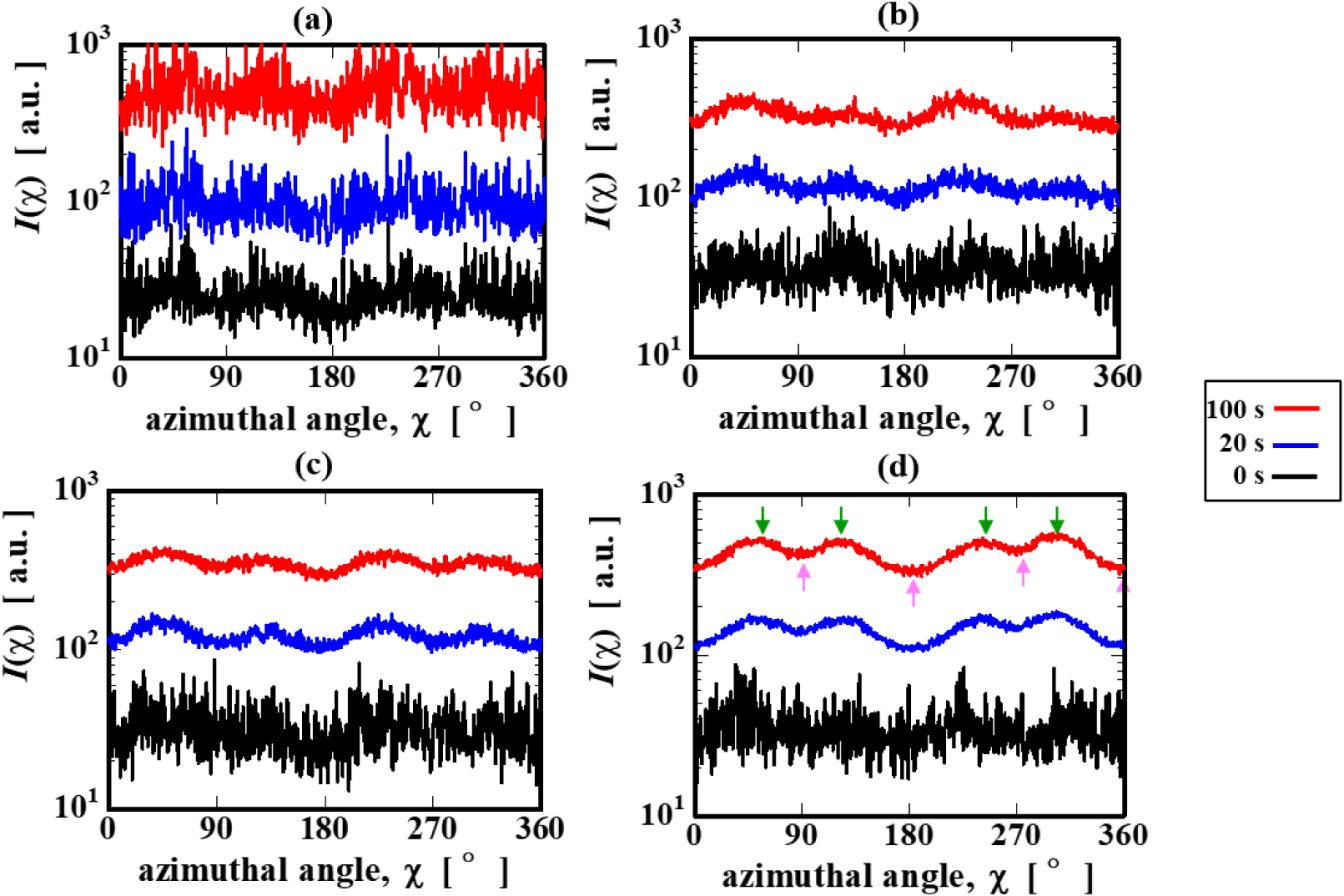
Azimuthal intensity profiles *I*(χ) extracted at *q* = 1.88 ± 0.04 μm^–1^ from the HV scattering patterns obtained at 0, 20, and 100 s under constant shear stresses of (a) 10 Pa, (b) 40 Pa, (c) 60 Pa, and (d) 200 Pa. The horizontal axis represents the azimuthal angle, χ, in degrees. The 4-fold anisotropy is characterized by intensity maxima near 45°, 135°, 225°, and 315°, and minima near 0°, 90°, 180°, and 270°. In panel (d), downward green arrows on the 100 s profile indicate the peak directions used to define the mean peak intensity, *I̅_max_*, whereas upward pink arrows indicate the trough directions used to define the mean trough intensity, *I̅_min_*.

To discuss the time evolution of the HV scattering more quantitatively, we used the mean peak intensity *I̅_max_*, the mean trough intensity *I̅_min_*, and the azimuthal modulation index C­(*t*), which represents the normalized peak-to-trough amplitude of the 4-fold pattern. Here, *I̅_max_* and *I̅_min_* denote the mean intensities at the fixed angular positions of the 4-fold pattern on the selected q annulus; they are not the integrated intensities of the full peaks or troughs over all angles. Because the angular positions of the maxima and minima remained nearly unchanged throughout the measurement, C­(*t*) provides a simple and direct measure of the temporal evolution of the 4-fold anisotropy in the present data set. As shown in Figure 10a, the average intensity at 45°, 135°, 225°, and 315° was defined as the mean peak intensity, *I̅_max_*, whereas, as shown in Figure 10b, the average intensity at 0°, 90°, 180°, and 270° was defined as the mean trough intensity, *I̅_min_*. As shown in Figures 10a and 10b, at 40, 60, and 200 Pa, *I̅_max_* decreased sharply from 0 to 20 s, whereas *I̅_min_* increased markedly. This behavior does not mean that the anisotropy itself became weaker. Rather, it indicates that, at the selected *q*, the angular intensity distribution broadened after stress application, so that intensity was redistributed from the nominal peak directions toward adjacent angles and even into the nominal trough directions. In other words, immediately after stress application, the HV pattern changed not only in amplitude but also in angular distribution, indicating that the 4-fold-symmetric pattern was reorganized rapidly. Thereafter, over 20–100 s, the changes in *I̅_max_* and *I̅_min_* became relatively gradual, suggesting that a quasi-steady anisotropic structure was maintained after the initial rapid change. These behaviors are in good agreement with the sharp initial decrease and subsequent gradual change in the characteristic correlation length observed in the HH scattering, as well as with the initial rapid deformation and the subsequent gradual deformation observed in the creep curves.

**Figure 10:**
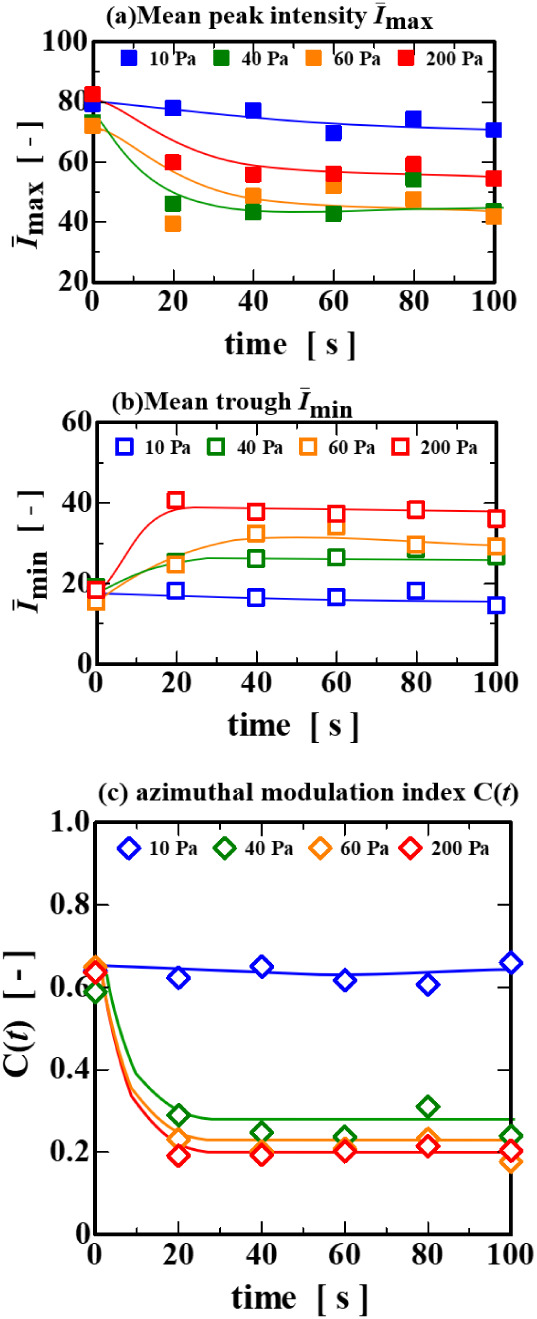
Time evolution of the quantitative parameters extracted from the HV azimuthal intensity profiles at *q* = 1.88 ± 0.04 μm^–1^: (a) mean peak intensity, *I̅_max_*, defined as the average intensity at the fixed angular positions 45°, 135°, 225°, and 315°; (b) mean trough intensity, *I̅_min_*, defined as the average intensity at 0°, 90°, 180°, and 270°; and (c) azimuthal modulation index, C­(*t*) =(*I̅_max_−I̅_min_*)/(*I̅_max_* + *I̅_min_*) and *I̅_min_* represent local mean intensities at these nominal peak and trough directions on the selected annulus, rather than integrated intensities of the full azimuthal lobes over their entire angular widths. The data show a rapid change in angular intensity distribution immediately after stress application, followed by a more gradual evolution at longer times.

The azimuthal modulation index, C­(*t*), shown in Figure 10c, decreased sharply over 0–20 s at 40, 60, and 200 Pa and then remained nearly constant after 20 s. Thus, the major changes in the angular distribution of the HV scattering also occurred predominantly immediately after stress application, similarly to those in the HH scattering. On the other hand, the value of C­(*t*) over 20–100 s tended to decrease in the order of 40, 60, and 200 Pa. This does not mean that the 4-fold-symmetric scattering pattern disappeared at higher stress; rather, it suggests that the angular modulation decreased because the scattering in the maximum directions became distributed over a wider angular range and extended into the minimum directions. Accordingly, a lower C­(t) at higher stress does not contradict the stress-enhanced structural anisotropy observed in the HH scattering; instead, it reflects broader angular spreading of the HV intensity at the selected *q*. In other words, under higher stress, the anisotropic reorganization of the CNF network progressed more strongly, while the angular distribution of the orientational correlation observed in the HV scattering became broader. Although the C­(*t*) value at 10 Pa is useful as a reference, it should not be interpreted on the same basis as those at 40–200 Pa because, as noted above, it may include an apparently high contrast under low-signal conditions.

Overall, the HV scattering analysis revealed that, in the CNF suspension, a 4-fold-symmetric pattern reflecting orientational correlation formed rapidly after constant stress application, and that an anisotropic structure with maintained principal axes was subsequently sustained. In particular, at 40–200 Pa, a decrease in the mean peak intensity, an increase in the mean trough intensity, and a rapid decrease in the modulation index were commonly observed over 0–20 s, confirming a two-stage response in the HV scattering as well, consisting of an initial rapid reorganization followed by a more gradual evolution. These results are consistent with the anisotropic mesoscale structural reorganization indicated by the HH scattering and demonstrate that combined HH/HV analysis by Rheo-SALS is effective for multifaceted evaluation of the structural reorganization and the time evolution of orientational correlation in the CNF network.

### Integrated Discussion with the Previous Polarized Imaging Results

The HH and HV scattering results obtained in the present study are in good agreement with the polarized imaging results previously reported using Rheo-Iris.^16^ This comparison provides an independent basis for evaluating the present in situ Rheo-SALS interpretation, because the Rheo-Iris measurements provide real-space information on birefringence and orientation axes, whereas the Rheo-SALS measurements provide reciprocal-space information on characteristic correlation length and orientational correlation. In the previous study, constant-stress creep tests were performed on the same mechanically fibrillated CNF suspension, and a rapid deformation immediately after stress application, followed by a gradual flow development, was observed under all stress conditions of 10, 40, 60, and 200 Pa. In the present Rheo-SALS measurements as well, at 40–200 Pa, the characteristic correlation length, ξ, obtained from the HH scattering, decreased sharply during 0–20 s, and the modulation index, C­(*t*), obtained from the HV scattering, also decreased rapidly within the same time window, followed by a more gradual evolution over 20–100 s. Therefore, both methods indicate that the major structural reorganization of the CNF network is concentrated immediately after stress application. The consistency between these independently obtained rheo-optical and scattering results supports the conclusion that the initial creep response is accompanied by rapid mesoscale structural reorganization and development of orientational correlation in the CNF network.

At 10 Pa, the changes in the HH scattering patterns and scattering profiles were small, and almost no directional difference in the characteristic correlation length was observed. In addition, the time variation of the 4-fold-symmetric pattern in the HV scattering was limited. In contrast, in the previous Rheo-Iris study, at 10 Pa and below, the retardation was low and uniform, and the orientation axes were randomly distributed, suggesting that an isotropically entangled CNF network structure was retained under quiescent conditions or under very small deformation.^16^ Therefore, under the low-stress condition, the real-space picture revealed by Rheo-Iris, namely, an isotropically entangled network, is mutually consistent with the reciprocal-space picture revealed by Rheo-SALS, in which little development of scattering anisotropy is observed.

Next, in the near-yield regime at 40–60 Pa, the HH scattering in the present study showed a sharp decrease in ξ during 0–20 s, followed by the appearance of a difference between the 0° and 90° directions. In the HV scattering, a 4-fold-symmetric pattern with intensity maxima at 45°, 135°, 225°, and 315° and intensity minima at 0°, 90°, 180°, and 270° was formed, and the angular modulation changed abruptly owing to the decrease in the mean peak intensity and the increase in the mean trough intensity. In the previous Rheo-Iris study, a spiral-shaped region with high retardation developed at around 40 Pa, and the orientation axes were distributed in the range of 60–85° with respect to the flow direction, that is, mainly toward the vorticity direction.^16^ Furthermore, at 60 Pa, a similar spiral-shaped, high-retardation pattern appeared at the initial stage, after which the retardation in the outer region decreased with time. Taken together, these results suggest that, near yielding, the initially coarse isotropic network first collapses and is reorganized on a smaller length scale, while, in real space, a log-rolling-like mesoscale structure oriented toward the vorticity direction develops, and, in reciprocal space, the corresponding anisotropic orientational correlation emerges.^15,21^ The 4-fold-symmetric pattern observed in the present HV scattering is consistent with the formation of the spatially heterogeneous oriented birefringence observed in the previous study.

In contrast, under the high-stress condition of 200 Pa, the anisotropy ratio, A_ξ_, obtained from the HH scattering became the largest, whereas the HV scattering retained the 4-fold-symmetric pattern itself but showed a lower value of the modulation index, C­(*t*), than those at 40 and 60 Pa. At first glance, this behavior may appear to indicate that the anisotropy becomes weaker at higher stress; however, when interpreted together with the previous Rheo-Iris results, the opposite is more likely to be the case. In the previous study, at 200 Pa, the retardation increased over the entire observation area, and the orientation axes were strongly aligned with the flow direction throughout the field.^16^ In addition, the log-rolling structure with vorticit*y*-direction alignment formed near the yielding regime was interpreted to collapse under high stress, leading to the alignment of smaller CNF aggregate units along the flow direction.^13^ Therefore, the decrease in the azimuthal modulation observed in the present HV scattering should not be interpreted as a loss of orientational correlation; rather, it is more reasonably explained by a broadening of the angular distribution, because the smaller structural units become distributed over a wider angular range and the scattering extends even into the minimum directions. In other words, the “more pronounced structural anisotropy at higher stress” indicated by the HH scattering, the “lower azimuthal modulation at higher stress” indicated by the HV scattering, and the “uniformly flow-aligned oriented birefringence” revealed by Rheo-Iris are all consistent with a common structural picture involving the subdivision of CNF aggregates and their alignment along the flow direction under high stress.

Overall, the previous Rheo-Iris study provides the spatial distributions of birefringence and orientation axes in real space, whereas the present Rheo-SALS study provides the time evolution of the characteristic correlation length and orientational correlation in reciprocal space. In this sense, the two methods are complementary. The former shows where and in what direction the oriented structures are formed, whereas the latter shows what representative length scale is reorganized and to what degree of anisotropy. Thus, by integration of the two methods, the creep process of the mechanically fibrillated CNF suspension can be understood as a stress-dependent hierarchical structural transition consisting of (i) the maintenance of an isotropically entangled network at low stress, (ii) the formation of a log-rolling-like mesoscale structure accompanied by vorticit*y*-direction alignment near yielding, and (iii) the collapse of that structure and the flow-direction alignment of smaller aggregate units at high stress. In principle, simultaneous rheo-SALS and rheo-Iris measurements in a single experiment would be a powerful approach because they could provide reciprocal-space scattering information and real-space birefringence/orientation information under exactly the same deformation history. However, such a combined experiment would require a dedicated optical design to integrate scattering detection and polarized imaging, synchronize the two detection systems, and control the polarization conditions without mutual optical interference. Therefore, the development of an integrated Rheo-SALS/Rheo-Iris platform is beyond the scope of the present study, which focuses on the reciprocal-space analysis of CNF structural reorganization using Rheo-SALS. Such an integrated approach or a broader multimodal rheo-optical study would be an interesting subject for future methodological work. Further comparison with additional external or complementary measurements would be valuable for deepening our interpretation. For example, standalone SALS or birefringence measurements on samples subjected to comparable processing histories could provide additional information about the persistence of anisotropic structures outside the Rheo-SALS configuration. In addition, complementary in situ techniques such as Rheo-IR, Rheo-NMR, or other rheo-spectroscopic methods could provide information on molecular interactions, hydration, or water dynamics and local fibril mobility during stress application, which cannot be obtained from SALS alone. Such multimodal characterization would enable a more detailed understanding of stress-induced internal structural changes across molecular, mesoscopic, and macroscopic length scales in CNF suspensions.

## Conclusions

In this study, Rheo-SALS measurements were performed on a mechanically fibrillated cellulose nanofibril (CNF) suspension, and the time evolution of the scattering patterns during constant-stress creep was analyzed in the HH and HV configurations at 10, 40, 60, and 200 Pa. These stress conditions span the preyield, near-yield, and postyield regimes of the suspension.

The HH scattering analysis showed that, at 40–200 Pa, the characteristic correlation length, ξ, decreased sharply during the first 0–20 s after stress application and then evolved more gradually, while the anisotropy ratio, A_ξ_, over 20–100 s increased with increasing stress and became largest at 200 Pa. The HV scattering analysis revealed a 4-fold-symmetric angular distribution with intensity maxima at 45°, 135°, 225°, and 315° and minima at 0°, 90°, 180°, and 270°. The mean peak intensity decreased, the mean trough intensity increased, and the modulation index, C­(*t*), decreased rapidly during 0–20 s, indicating that the major structural reorganization was concentrated immediately after stress application. At higher stress, the lower C­(*t*) values suggest a broader angular distribution of orientational correlation rather than a loss of anisotropy.

Together with the previous Rheo-Iris results, these findings indicate that the creep process of the mechanically fibrillated CNF suspension can be understood as a stress-dependent hierarchical structural transition: maintenance of an isotropically entangled network at low stress, formation of a log-rolling-like mesoscale structure with vorticit*y*-direction alignment near yielding, and collapse of that structure together with flow-direction alignment of smaller aggregate units at high stress. Thus, combined HH/HV Rheo-SALS analysis provides an effective framework for quantitatively describing structural reorganization and orientational correlation in reciprocal space and for advancing the understanding of process–structure–property relationships in CNF suspensions. From a process-structure–property viewpoint, these results indicate that the imposed stress level and the early-stage creep history determine the structural pathway of the CNF network, which in turn governs the macroscopic deformation response and the type of flow-induced anisotropy that develops. In particular, the present Rheo-SALS analysis reveals that the dominant micro/mesoscale reorganization is concentrated within the first 0–20 s after stress application, providing a process-relevant time window for controlling structural evolution in CNF suspensions. This point is potentially important for designing processing conditions that either preserve a more isotropic network structure or promote controlled anisotropic organization, depending on the targeted CNF processing route and material function. More generally, the present approach should be applicable to other optically accessible soft materials and complex fluids in which deformation or flow induces changes in mesoscale heterogeneity, characteristic structural length scale, anisotropy, or orientational correlation. Examples include dispersed, network-forming, or self-assembled systems whose nonlinear rheological behavior is governed by flow-induced structural reorganization. Therefore, Rheo-SALS can serve as a general reciprocal-space tool for linking macroscopic rheological response to evolving internal structure under processing-relevant deformation histories. It should be noted, however, that the present measurements were conducted at a single CNF concentration and temperature. Both parameters are expected to influence the quantitative rheological and SALS responses. Increasing the CNF concentration would likely enhance interfibril interactions, network connectivity, and structural heterogeneity, thereby affecting the apparent yielding behavior, characteristic correlation length, scattering intensity distribution, and degree of shear-induced anisotropy. Temperature may also affect the response through changes in the viscosity of the continuous phase, thermal motion, relaxation dynamics, and the balance of interactions within the CNF network. Therefore, systematic Rheo-SALS measurements over a wider range of CNF concentrations and temperatures will be an important subject for future work to establish the generality of the present observations and further clarify the structure–rheology relationship in CNF suspensions.

## Supplementary Material



## Data Availability

All data generated or analyzed during this study are included in this published article and its Supporting Information.

## Notes

q-Ranges used to determine the characteristic scattering vector q*; summary of q* values obtained from the intersections of the low-q and high-q linear fits (PDF)
