# Supplementary material for: Reciprocal-Space Analysis of Structural Reorganization and Orientational Correlation in a Cellulose Nanofibril Suspension during Constant-Stress Creep
Source: Langmuir. 2026 Jul 24;42(31):22799–811. doi: 10.1021/acs.langmuir.6c02557 (PMC13472186; doi:10.1021/acs.langmuir.6c02557)
Supplement: Supplementary file 1 [file la6c02557_si_001.pdf]

Supporting Information

Reciprocal-Space Analysis of Structural Reorganization and Orientational Correlation in a Cellulose Nanofibril Suspension during Constant-Stress Creep

*Yoshifumi Yamagata[1, 2], Moe Araida[1], Saki Otobe[1]

[1] Business Unit Characterization, Anton Paar Japan K. K.
1st fl., Riverside Sumida, Tsutsumi-dori 1-19-9, Sumida-ku, Tokyo 131-0034, Japan

[2] Research Institute for Science and Technology, Tokyo University of Science
2641 Yamazaki, Noda-shi, Chiba, 278-8510, Japan

*Corresponding author:
Yoshifumi Yamagata e-mail: yoshifumi.yamagata@anton-paar.com
Tel: +81-80-2174-4840
ORCID ID: 0000-0002-2193-887X

Number of pages: 2
Number of figures: 0
Number of schemes: 0
Number of tables: 2

Table of Contents

Table S1. q-ranges used for determination of the characteristic scattering vector q^*. For each stress and azimuthal direction, the same fitting windows were used for all time points. Data points possibly affected by detector saturation in the lowest-q region were excluded from the fitting.

Table S2. Summary of the characteristic scattering vector $q*$ determined from the intersection of the linear fits in the low-q and high-q regions.

Table S1. q-ranges used for determination of the characteristic scattering vector q*. For each stress and azimuthal direction, the same fitting windows were used for all time points. Data points possibly affected by detector saturation in the lowest-q region were excluded from the fitting.

shear stress [Pa]	direction [°]	time [s]	low-q fit [μm^{-1}]	high-q fit [μm^{-1}]
10	0	0 ~ 100	0.35 ~ 0.80	1.20 ~ 3.20
	90	0 ~ 100	0.30 ~ 0.85	1.20 ~ 3.20
40	0	0 ~ 100	0.35 ~ 0.85	1.20 ~ 3.00
	90	0 ~ 100	0.35 ~ 0.90	1.20 ~ 3.00
60	0	0 ~ 100	0.35 ~ 0.85	1.20 ~ 3.00
	90	0 ~ 100	0.30 ~ 0.90	1.20 ~ 3.00
200	0	0 ~ 100	0.35 ~ 0.70	1.00 ~ 3.00
	90	0 ~ 100	0.30 ~ 0.85	1.10 ~ 3.00

Table S2. Summary of the characteristic scattering vector q∗ determined from the intersection of the linear fits in the low-q and high-q regions.

	10 Pa		40 Pa		60 Pa		200 Pa	
	0°	90°	0°	90°	0°	90°	0°	90°
0 s	0.620	0.620	0.575	0.657	0.690	0.694	0.547	0.571
20 s	0.882	0.882	1.057	0.984	1.000	1.106	0.731	1.069
40 s	0.931	0.931	1.053	1.114	1.000	1.110	0.706	1.057
60 s	0.939	0.939	1.061	1.106	1.033	1.069	0.694	1.069
80 s	0.931	0.931	1.046	1.138	1.008	1.077	0.706	1.069
100 s	0.927	0.927	1.041	1.158	1.012	1.097	0.690	1.069
